# Supplementary material for: Development of a computer-aided design software for the quantitative evaluation of aesthetic damage
Source: PLoS One. 2019 Dec 18;14(12):e0226322. doi: 10.1371/journal.pone.0226322 (PMC6919621; doi:10.1371/journal.pone.0226322)
Supplement: S1 Code site — 1. (ZIP) [file pone.0226322.s001.zip › [EESC_jr][Projeto]Relatorio_07_21/reports/templates_zip/gentelella-master/vendors/Flot/examples/axes-multiple/index.html]

Flot Examples: Multiple Axes


## Multiple axes

Multiple axis support showing the raw oil price in US $/barrel of crude oil vs. the exchange rate from US $ to €.

As illustrated, you can put in multiple axes if you need to. For each data series, simply specify the axis number. In the options, you can then configure where you want the extra axes to appear.

Position axis left or right.

Copyright © 2007 - 2014 IOLA and Ole Laursen
